# Commentary on: Cross-linked Hyaluronic Acid for Cleft Lip and Palate Aesthetic Correction: A Preliminary Report

**DOI:** 10.1093/asjof/ojac069

**Published:** 2022-08-18

**Authors:** Christopher A Derderian

**Affiliations:** Associate professor, Department of Plastic Surgery, UT Southwestern, Dallas, TX, USA

This paper presents a case series of 15 patients treated with hyaluronic acid (HA) fillers of varying degrees of cross-linkage for aesthetic concerns of the lip and nose due to cleft lip and palate.^1^ The average volume of the filler was 4.2 mL per patient (range 2-10 mL). The majority of patients were treated in 1 or 2 sessions, but 1 patient had 5 sessions separated by 4-6 weeks (Video).

Persistent asymmetries of the lip and nose due to cleft lip and palate present significant reconstructive challenges. Insufficient volume of the upper lip is commonly observed in the presence of a satisfactory scar and adequate vermilion height at the site of the cleft lip repair. The lip may appear deflated and/or flat rather than convex, particularly in patients with bilateral cleft palate. Dermal fat grafting and/or fat grafting have been the predominant approach to correct the observed volume deficiency of the upper lip. There are a number of common features of the cleft lip nasal deformity but nasal asymmetry is the dominating feature that stigmatizes patients. The bony pyramid and nasal dorsum are typically deviated away from the side of the cleft in the unilateral cleft lip. In addition, there is asymmetry of the nasal tip and alae. In the bilateral cleft lip, the nose is typically wide and the nasal tip is bulbous, over-rotated, and under-projected.

As pointed out by the author, these patients frequently express fatigue at the number of surgeries that they have had over their lifetime and this may lead them to consider nonsurgical alternatives to improve their appearance. Fillers have the advantage of minimal downtime and recovery. The obvious downside of fillers is the durability of volume augmentation and long-term cost associated with the expected need for retreatment. The author was also careful to highlight the potential complications associated with filler injections that can include skin necrosis, particularly when injecting the nose in the presence of excessive scarring. In this study, a blunt cannula was always used for nasal injections.

The author shows a number of cases that demonstrate a remarkable improvement in the appearance of the lip and nose with treatment. The author also reported that patients had a high degree of satisfaction at the end of treatment, immediately after completion of injecting HA.

This study suffers from a small sample size and heterogeneity of the patient population with regard to age, gender, and type of cleft lip. However, there is convincing qualitative evidence that there may be a role for HA filler in improving the appearance of both the lip and nose for patients with residual deformities of the lip and nose due to cleft lip. How expansive that role should be is the product of a detailed and transparent discussion of the patient’s goals and the setting of patient expectations for what can be achieved with fillers, including both durability and long-term cost.

Although this was not assessed in the study, the case photos demonstrate that the lip is more amenable to change with HA fillers than the nose. The initial improvements in the appearance of the upper lip were on par with those achieved by fat grafting and/or dermal fat graft. The current paper suggests that the patient with residual stigmata of cleft lip in the lip and nose who are seeking improvement without surgery should consider HA filler.

## Supplementary Material

ojac069_Supplementary_Data
